# Supplementary material for: Tissue Sheet Engineered Using Human Umbilical Cord-Derived Mesenchymal Stem Cells Improves Diabetic Wound Healing
Source: Int J Mol Sci. 2022 Oct 21;23(20):12697. doi: 10.3390/ijms232012697 (PMC9604116; doi:10.3390/ijms232012697)
Supplement: Supplementary file 1 [file ijms-23-12697-s001.zip › ijms-1969967-supplementary.pdf]

Supplementary Information

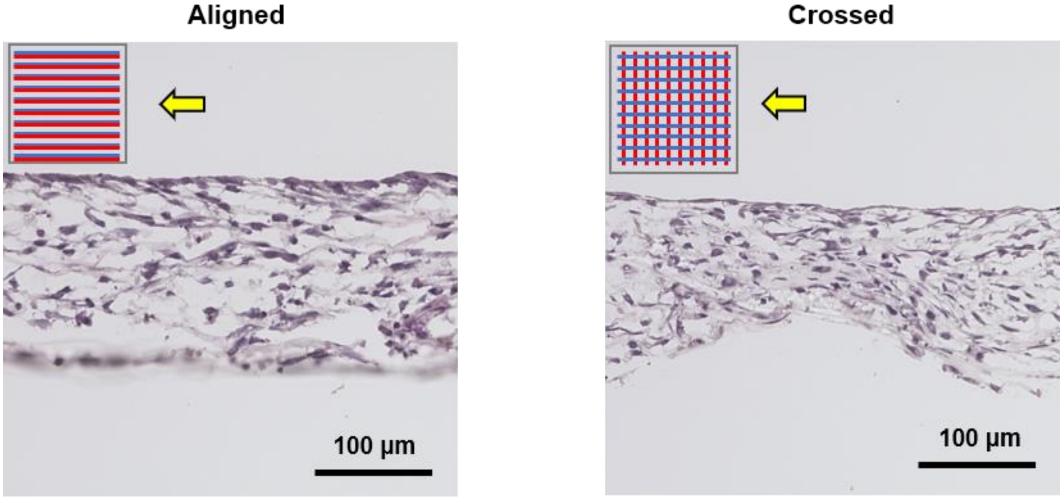

Figure S1. Tissue sheet formation of human umbilical cord mesenchymal stem cell (hUC-MSC) using poly(lactide-co-glycolide) scaffold. Hematoxylin and eosin staining of hUC-MSC tissue sheets with different fiber structures (aligned and crossed). Scale bar = 100 μm. The yellow arrowheads show a schematic of the two types of PLGA fiber structures.

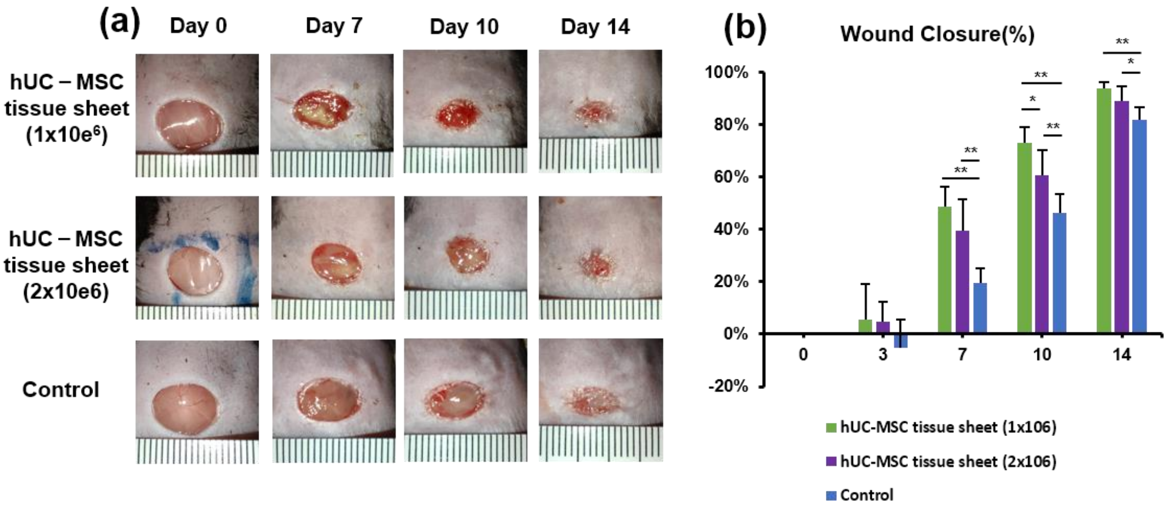

Figure S2. Tissue sheets with different cell numbers promote wound healing. (**a**) Representative photographs of full-thickness excision wounds on days 0, 7, 10, and 14 after wounding. (**b**) Quantitative analysis of the rate of wound closure in each group. Significance was determined using analysis of variance ANOVA. * $p < 0.05$, ** $p < 0.01$.
